# The Spectrum of Neurological and Sensory Abnormalities in Gaucher Disease Patients: A Multidisciplinary Study (SENOPRO)

**DOI:** 10.3390/ijms24108844

**Published:** 2023-05-16

**Authors:** Maria Giulia Tullo, Emanuele Cerulli Irelli, Francesca Caramia, Gianmarco Tessari, Carlo Di Bonaventura, Rosaria Turchetta, Anna Teresa Giallonardo, Giovanna Palumbo, Simona Bianchi, Francesca Atturo, Marcella Nebbioso, Patrizia Mancini, Cecilia Guariglia, Fiorina Giona

**Affiliations:** 1 Department of Translational and Precision Medicine, “La Sapienza” University of Rome, 00161 Rome, Italy; mariagiulia.tullo@uniroma1.it (M.G.T.);; 2 Department of Neuroscience, Imaging and Clinical Sciences, ITAB—Institute of Advanced Biomedical Technologies, “G. D’Annunzio” University, 66100 Chieti, Italy; 3 Department of Human Neuroscience, “La Sapienza” University of Rome, 00185 Rome, Italy; emanuele.cerulliirelli@uniroma1.it (E.C.I.);; 4 Department of Psychology, “La Sapienza” University of Rome, 00185 Rome, Italy; 5 PhD Program in Behavioral Neuroscience, “La Sapienza” University of Rome, 00185 Rome, Italy; 6 Department of Sense Organs, “La Sapienza” University of Rome, 00185 Rome, Italy; 7 Cognitive and Motor Rehabilitation and Neuroimaging Unit, IRCCS Fondazione Santa Lucia, 00179 Rome, Italy

**Keywords:** Gaucher disease, Parkinson’s disease, audiometric, hearing loss, evoked potentials, cognitive, psychiatric, sleep

## Abstract

Gaucher disease (GD) has been increasingly recognized as a continuum of phenotypes with variable neurological and sensory involvement. No study has yet specifically explored the spectrum of neuropsychiatric and sensory abnormalities in GD patients through a multidisciplinary approach. Abnormalities involving the nervous system, including sensory abnormalities, cognitive disturbances, and psychiatric comorbidities, have been identified in GD1 and GD3 patients. In this prospective study, named SENOPRO, we performed neurological, neuroradiological, neuropsychological, ophthalmological, and hearing assessments in 22 GD patients: 19 GD1 and 3 GD3. First, we highlighted a high rate of parkinsonian motor and non-motor symptoms (including high rates of excessive daytime sleepiness), especially in GD1 patients harboring severe glucocerebrosidase variants. Secondly, neuropsychological evaluations revealed a high prevalence of cognitive impairment and psychiatric disturbances, both in patients initially classified as GD1 and GD3. Thirdly, hippocampal brain volume reduction was associated with impaired short- and long-term performance in an episodic memory test. Fourthly, audiometric assessment showed an impaired speech perception in noise in the majority of patients, indicative of an impaired central processing of hearing, associated with high rates of slight hearing loss both in GD1 and GD3 patients. Finally, relevant structural and functional abnormalities along the visual system were found both in GD1 and GD3 patients by means of visual evoked potentials and optical coherence tomography. Overall, our findings support the concept of GD as a spectrum of disease subtypes, and support the importance of in-depth periodic monitoring of cognitive and motor performances, mood, sleep patterns, and sensory abnormalities in all patients with GD, independently from the patient’s initial classification.

## 1. Introduction

Gaucher Disease (GD) is an autosomal recessive metabolic disorder due to glucocerebro-sidase (glucosylceramidase, or acid beta glucosidase—GBA) deficiency, caused by GBA 1q21 genetic mutation. As a consequence, the glucosylceramide (or glucocerebrosidase) accumulates in the liver, the spleen, and the bone marrow endothelial reticulum cells [1]. GD incidence is estimated to be 1:60.000 worldwide, but much higher (1/1.000) in Ashkenazi Jews. Clinical symptoms are extremely variable. Classically, three main phenotypes of GD are recognized based on the neurological involvement and the severity of the disease. Type 1 GD (GD1), the most common subtype, is traditionally considered non-neuropathic. However, neurological symptoms can appear during the course of the disease. Parkinsonism is the most common neurological complication that can occur in patients with a variety of the approximately 600 GD-causing GBA mutations in the heterozygous state [2]. Type 2 GD (GD2) is the acute neuropathic form, which manifests as a severe and early involvement of the Central Nervous System (CNS), usually within the first few months after birth. Symptoms may include supranuclear gaze palsy, epilepsy, and progressive spasticity. Nearly all GD2 children do not survive beyond the age of two or three years. Type 3 GD (GD3) is characterized by a chronic neurological involvement, ranging from supranuclear gaze palsy, slowly progressive dementia, ataxia, spasticity, and epilepsy. The risk of neuronopathic involvement has been associated with the patient’s genotype, with more severe variants (e.g., p.L483P, previously known as L444P, and p.A448H, previously known D409H) conferring an increased chance of developing neurological subtypes of GD compared with milder variants (p.A409S, previously known as N370S) [3,4,5]. However, this classification shows some limitations since there is a wide spectrum of disease manifestations and disease severity within the different types [6]. Increasing evidence has shown that GD1 patients may have progressive neurological manifestations, including Parkinson’s disease (PD) or Parkinsonisms, even in those with mutations considered protective of neurological involvement, such as N370S (p.A409S) in a heterozygous state [7]. Recently, it was reported that genetic mutations included in the PD risk score were more frequent in GD1 patients who developed PD or Parkinsonisms, suggesting that common mutations can influence underlying biological pathways [2]. Studies have highlighted that patients can move from the GD1 to GD3 subtype during the course of the disease and that careful neurological examinations can often lead to reconsidering the GD subtype classification [7,8,9]. For this reason, GD should be considered as a continuum of phenotypes as some patients may not be definitively included in one of the three subtypes [6]. Over the past few years, a wide range of abnormalities involving the nervous system, including sensory abnormalities (i.e., visual and hearing impairment), structural brain abnormalities [10], and cognitive disturbances and psychiatric comorbidities [11,12,13,14] have been identified in GD1 and GD3 patients. Although cognitive deficits are well known in Gaucher type 3 [15], a few studies assessed cognitive evaluations in GD1 patients [16,17,18]. Minor deficits in focusing attention and a slow retrieval of information held in memory, without influencing daily activity, have been found in GD1 patients [17].

In spite of these observations, no comprehensive multidisciplinary studies to thoroughly explore the spectrum of CNS and sensory involvement in GD patients have yet been published.

Based on this background, we planned a prospective study with the aim to investigate the CNS and sensory disturbances in GD1 and GD3 patients in depth through an extensive multidisciplinary evaluation to identify clinical and subclinical neurological manifestations, including cognitive impairment and behavioral alterations. Here, we report the results of the baseline investigations, including neurological, neuroradiological, neuropsychological, ophthalmological, and hearing assessments of 22 GD patients.

## 2. Results

### 2.1. Clinical Features

Twenty-two GD patients (nineteen GD1 and three GD3; nine males and thirteen females) with a median age at the time of study of 44.5 years (range 18–68 years) were studied between June 2019 and February 2020. As shown in Table 1, all but one of the nineteen GD1 patients (95%) had the p.A409S (previously known as N370S) mutation, combined with p.L483P (previously known as L444P) in eight (44%). At the time of the enrolment in the study, all GD3 patients presented ocular movement abnormalities, combined with muscle weakness in one patient; in addition, 3/19 GD1 patients had neurological or neuropsychological signs (one Parkinson disease, one cognitive impairment, one psychomotor slowing). At the time of the study evaluation, all GD1 patients, except one, had been in treatment for a median of 17.3 years.

### 2.2. Neurological Findings

All 22 patients underwent neurological assessment; however, brain MRI was performed in only 19/22 (17 GD1) patients due to claustrophobia. The results of neurological, neuroradiological, and sensory evaluations in each patient are detailed in Table 2. Abnormal saccadic movements were found in all GD3 patients and in one GD1 patient (pt #01). Mild parkinsonian motor signs were found in 7/19 GD1 patients (37%), including one patient with a previous diagnosis of Parkinson disease (PD) (pt #04, as mentioned above). Out of these seven patients, three had a combination of bradykinesia and rigidity, and one patient (pt #04) had rest tremor. The median age was higher among patients with parkinsonian motor signs (46 years, interquartile range (IQR) 32–49) compared with the others (42 years, IQR 28-53) (*p* = 0.3).

**Figure 1:**
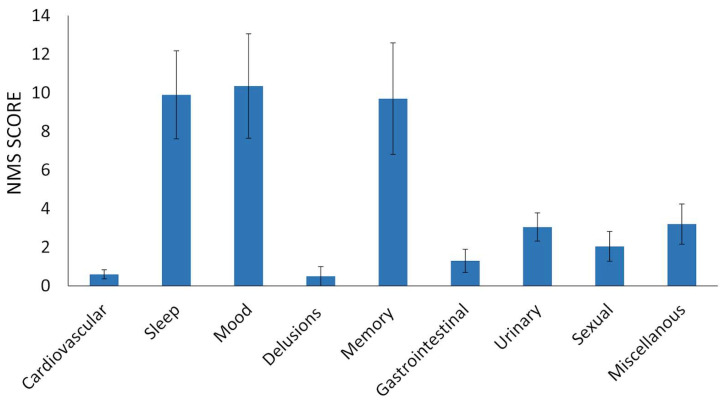
Non-motor symptom (NMS) scores.

Considering the entire cohort, the mean of the Movement Disorder Society-Unified Parkinson’s Disease Rating Scale part III (MDS-UPDRS III) score was 2.91 (SD ± 6.1). The non-motor symptom scale (NMSS) revealed a mean non-motor symptoms (NMS) score of 38.8 (SD ± 32.6), with the highest scores observed in the sleep/fatigue domain, mood/apathy domain, and memory/attention domain (Figure 1). Total NMS score was significantly correlated with total MDS-UPDRS III score (rho = 0.54, *p* = 0.01) as well as NMS memory/attention scores (rho = 0.676, *p* = 0.001).

The Epworth Sleepiness Scale (ESS) led us to discover excessive daytime sleepiness (EDS) in 9/22 (41%) patients (8 GD1), with a mean ESS score of 8.2 (SD ± 5). Due to a severe EDS and daily episodes of sleep attacks, one patient (pt #19) performed a battery of advanced tests (multiple sleep latency test, polysomnography, measurement of cerebrospinal fluid Orexin, HLA test) to rule out narcolepsy and sleep apnea, and was, then, diagnosed with central hypersomnia. In the entire cohort, fatigue scores measured using NMSS were found to be significantly associated with ESS scores (r = 0.48, *p* = 0.04). Regarding other sleep disturbances, five GD1 patients (23%) were diagnosed with restless leg syndrome and a possible rapid-eye-movement (REM) behavior disorder was found in another three GD1 patients. Using dedicated items, insomnia was detected in ten GD1 patients (45,45%), mild in six and moderate/severe in four patients.

The video electroencephalography (video-EEG) revealed a mild slowing of background activity in 2 GD3 patients and epileptiform abnormalities in one GD3 patient.

### 2.3. Magnetic Resonance Imaging (MRI) Findings

MR examinations revealed unspecific abnormalities in 8/19 evaluable patients (42%), (7/17 GD1 and 1/2 GD3). In particular, cortical and/or subcortical areas of gliosis (three GD1 patients), developmental venous anomaly extended from the left frontal surface of the brain to the lateral ventricle (one GD1 patient), dilation of perivascular spaces in the sub-cortical and nucleus–basal area (one GD1 patient), diffuse suffering of the brain white matter due to chronic ischemic vascular damage (one GD1 patient), and mild dilation of the posterior horns of the lateral ventricles (one GD1 and one GD3 patient) were found.

Statistical analysis on the hippocampal (HC) grey matter volume estimation was performed comparing MRI findings obtained in 19 GD patients (left HC: mean = 2.90, SD = 0.34; right HC: mean = 3.32, SD = 0.40) and in 19 healthy volunteers (left HC: mean = 2.87, SD = 0.27; right HC: mean = 3.27, SD = 0.31).

After controlling for age and total intracranial volume (TIV), the ANCOVA revealed a non-significant main effect of the group (healthy volunteers vs. GD patients) on hippocampal volume of neither the left (F(1,34) = 1.71, *p* = 0.20, partial eta-square = 0.04) nor the right (F(1,34) = 2.59, *p* = 0.11, partial eta-square = 0.07).

To investigate the association between hippocampal volume and neuropsychological deficit, we performed a partial correlation analysis. After controlling for age, schooling, and total intracranial volume, the analysis showed that the left hippocampal volume was positively correlated with the Babcock story test for both the immediate (rho = 0.55, *p* = 0.024) and the delayed part (rho = 0.69, *p* = 0.004).

### 2.4. Neuropsychological Findings

Complete neuropsychological batteries were performed in 20/22 patients (seventeen GD1, mean years of education = 11.64, SD ± 3.48; three GD3, mean years of education = 10.66, SD ± 4.61). Two GD1 patients (pts #03 and #15) did not complete the evaluation due to severe cognitive impairment (Mini-Mental State Examination (MMSE) scores were 19 and 9, respectively).

Among seventeen GD1 patients, three (pts #04, #11, #14) presented cognitive impairment in the memory domain; in one patient (pt #04), this was associated with attention domain deficit. Among three GD3 patients, one patient (pt #20) presented cognitive impairment in all neuropsychological domains (language, attention, memory, visuoconstruction, and executive functions) and one patient (pt #22) presented cognitive impairment in the memory domain.

Out of the twenty-two patients who underwent psychiatric evaluations using Brief Psychiatric Rating Scale (BPRS), five GD1 patients (23%) presented anxiety symptoms, combined with depression symptoms in one (pt #08), somatic concerns in one (pt #11), and depression and somatic concerns in one (pt #04). One GD1 patient (pt #18) had isolated depression symptoms, and four patients, one GD3 (pt #22) and three GD1, presented isolated somatic concerns. One GD1 patient (pt #03) had psychotic symptoms requiring drug treatment.

### 2.5. Hearing Findings

Out of the twenty-two patients, 13/19 GD1 (68%) and all three GD3 patients showed slight or moderate sensorineural hearing loss (SNHL); 4/13 GD1 patients had a cookie and reverse cookie bite morphology. Concerning hearing loss (HL) severity, on average, patients presented a mild down-sloping HL with pure tone audiometry (PTA) for the different frequency ranges of 17.4 dB HL (SD = 10.7) per 125–250 Hz; 19.3 dB HL (SD 18.4) per 500–2000 Hz; and 26.7 dB HL (SD 18.4) per 4000–8000 Hz.

Average speech perception for sentences in quiet was 98% (SD = 6.3), average Matrix score was −2.9 dB SNR (SD = 7.6) while the average V auditory brainstem response (ABR) wave threshold was 33.8 dB HL (SD = 18.2). Although the majority of patients (91%) presented with normal speech perception in a quiet setting (100% score), 91% showed a reduction in comprehension of speech perception in noise as a measure of a slight to moderate central processing deterioration in hearing.

Concerning the bivariate analysis of correlation between outcomes, as it might be expected, there is a correlation between PTA in the 4000–8000 Hz and ABR V wave threshold (rho 0.78; *p* < 0.001). Moreover, a correlation between Matrix test scores with PTA was found in the high frequency range (rho 0.6; *p* = 0.003).

### 2.6. Ophthalmological Findings

Ophthalmological evaluation highlighted a normal best-corrected visual acuity (BCVA) and intraocular pressure measurement (IOP) in all 22 patients. Superficial corneal dystrophies were observed in three patients (two GD1 and one GD3), while some degree of vascular tortuosity was observed at the fundus in almost all patients. In particular, the vascular tortuosity was more evident in 2/19 GD1 and 2/3 GD3 patients. Visual evoked potentials (VEPs) showed an increased latency and/or moderate reduction in amplitude of optic nerve function in 12/22 patients (54.5%) (3 GD3). Among the 44/22 eyes/patients, VEPs were normal in 20 eyes and altered in 24 eyes (54.5%); in particular, ten eyes had a reduction in amplitude (eight eyes of four GD1 patients, two eyes of one GD3 patient), six eyes had a reduction in amplitude and increase in latency (all GD1), and eight eyes had an increase in latency (four eyes of two GD1 and four eyes of two GD3 patients). The Spectral Domain-Optical Coherence Tomography (SD-OCT) examination showed impairment in retinal nerve fiber layers (RNFL) in 7/22 (32%) patients (six GD1), and this was combined with macular damage in two patients (pts #14 and #22). The full-field electroretinogram exam (ERG) was found to be altered in both eyes of one GD1 patient (pt #19) with a reduction in amplitude of single flash and flicker cone responses.

### 2.7. Neurological and Sensory Abnormalities According to Genotype

All patients (two with p.L483P/p.L483P and one with p.A448H/p.A448H mutations) diagnosed as GD3 at baseline showed a lower age at study evaluation compared with heterozygous patients diagnosed as GD1 at baseline (28 years, IQR 19–28 vs. 46 years, IQR 33–56; *p* = 0.053). Considering the neurological and sensory abnormalities observed in GD1 and GD3 patients, the only significant difference was the higher rate of saccadic impairment among the homozygous patients compared with the remaining cohort of heterozygous patients (3/3, 100% vs. 0/19: 0%; *p* < 0.001). When only considering heterozygous GD1 patients, a significantly higher rate of parkinsonian motor signs was found among p.L483P heterozygous patients compared with the remaining heterozygous patients (5/8, 62.5% vs. 2/11, 18.2%; *p* = 0.048).

## 3. Discussion

This is the first extensive multidisciplinary prospective study to evaluate CNS and sensory abnormalities in a cohort of GD patients. As expected, patients classified as GD3 at baseline were found to be more diffusely impaired, though all examined GD1 patients displayed at least one or more relevant dysfunctions. In this study, we found that 31.8% of patients show subtle parkinsonian motor signs, especially bradykinesia and rigidity, which were found to be more common among GD1 patients. As previously identified by Cherin and collaborators [19], our findings confirm that an extensive neurological examination could highlight parkinsonian motor signs in a relevant percentage of patients. Interestingly, a significant association between p.L483P heterozygous state and parkinsonian motor signs was found, supporting a deleterious effect of severe GBA mutations on the occurrence of neurological abnormalities (especially parkinsonism) in GD1 patients [20]. Moreover, we assessed non-motor symptoms through a dedicated scale developed for PD patients (i.e., NMSS), revealing a high burden of sleep, cognitive, and mood disturbances, as previously suggested by other studies using other scales [21]. Non-motor symptoms represent a core clinical finding in PD and may precede the onset of motor symptoms by many years [22]. Due to the general lack of studies specifically investigating the association between non-motor symptoms and motor signs in GD patients, it was not possible to rule out whether the non-motor symptoms were attributed to factors independent from PD (depressive symptoms related to concerns for a lifelong disease, occurrence of bone pain, periodic infusions of intravenous drugs, etc.). Notably, we found a significant correlation between the motor and non-motor scores in our patients, supporting the idea of shared underlying pathological mechanisms in GD patients. Pathological daytime sleepiness was found in approximately half of our cohort along with other sleep disturbances, including restless leg syndrome and REM behavior disorder. The high prevalence of sleep disturbances in GD patients observed in our multidisciplinary study, in line with the results of Wilke and co-authors [21], represent a relevant finding for GD clinical management, considering the impact of sleep disturbances on mood and quality of life [23]. Dedicated polysomnographic studies should be performed to better address the exact pathophysiology of sleepiness in GD. In fact, the observation of a severe central hypersomnia in one of our patients could also lead to speculation about the existence of a primary sleep disorder in GD, as observed in other lysosomal diseases [24,25].

Cognitive and psychiatric evaluations in all patients showed that cognitive impairment was more frequent in patients diagnosed as GD3 at baseline, as expected. When considering the patients initially diagnosed as GD1, a variable degree of cognitive disturbances—ranging from mild cognitive impairment to dementia—was found in approximately one fourth of them. Memory and attention were found to be the cognitive domains most frequently impaired, as previously reported [16,17,18]. However, the rate of GD1 patients with cognitive impairments in our study was higher when compared to the previous ones [16,17,18], supporting the importance of an in-depth approach in the evaluation of cognitive functioning both in GD1 and GD3 patients. For the first time, a significant association between hippocampal grey matter volume and memory deficit in GD1 and GD3 patients was found. Specifically, left hippocampal GM volume reduction was associated with impaired short- and long-term performance in an episodic memory test (Babcock test, a story recall test). It is known that medial temporal lobe (MTL) structures, such as the hippocampus, are involved in the formation of new memories. As a matter of fact, the association between hippocampal size and memory performance has been shown by several neuroimaging studies in other conditions [26,27]. In addition, atrophy of MTL structures has been found to be associated with episodic memory impairment, especially in neurocognitive diseases [28,29]. In our study, we found that the GM volume reduction was associated with memory impairment in the left, but not in the right, hippocampus. A large number of brain lesion and functional neuroimaging studies have shown that memory function is lateralized based on the material type. Indeed, left hemisphere structures (including left hippocampus) are implicated in verbal memory processing, while right hemisphere structures (including right hippocampus) support non-verbal/spatial memory [30,31]. Our results pave the way to future research about the association between cortical and subcortical brain changes and neuropsychological deficit in GD patients.

In our study, psychiatric evaluations revealed relevant symptoms in almost half of GD1 patients, especially anxiety and depression. To date, it is unclear whether the cognitive dysfunctions in GD patients are related to the disease or to an early phase of neurodegenerative disorder, such as PD. However, routine monitoring of cognitive performance, mood, behavior, and sleep patterns could be useful as early biomarkers for developing neurodegenerative disorders in GD patients.

With regard to hearing abnormalities, we documented for the first time a high prevalence of SNHL in GD1 patients. Previous studies mainly reported a wide range of abnormalities in GD3 patients [11] and only a few case reports focused on GD1 [13]. Interestingly, we also highlighted an altered speech perception in noise in almost all patients, including those without an underlying hearing loss. Altered speech perception in noise has been previously associated with other different neurological conditions, such as HIV-related neurocognitive disorders, depression, traumatic brain injury, and attention deficit hyperactivity disorders, suggesting impaired central processing of auditory information as the main pathophysiological process [32]. Importantly, we also highlighted the occurrence of a cookie-bite morphology in four patients (three of whom were diagnosed as GD3), which has been typically associated with genetic etiologies of hearing loss [33]. In this setting, our observation prompts future studies aiming to investigate whether the associated hearing loss was not only determined by the glucosylceramide accumulation but also to a coexisting functional impairment due to GBA mutations.

Non-invasive visual imaging assessments are becoming important tools in neurology to detect subclinical CNS involvement and early neurodegenerative processes [34]. In particular, VEPs have been used to assess modifications of the visual pathway integrity and visual cortex functioning during normal aging and neurodegeneration [35], whereas retinal thinning detected by SD-OCT has been considered an early marker of neurodegeneration in PD and in other neurodegenerative disorders [36]. In this study, VEPs were able to confirm a high rate of abnormalities both in GD1 and GD3 [37], and a thinning of retinal nerve fiber layers was observed in almost one third of patients. Previous studies highlighted a significant correlation between the thickness of the retinal layer, and both neurodegenerative markers and GD severity scores [38,39], suggesting that SD-OCT may be used as an early prognostic indicator of late neurological involvement in GD.

Our findings confirm that a thorough multidisciplinary approach could reveal a wide range of relevant disturbances, both in patients diagnosed with GD1 and GD3. The occurrence of at least one severe mutation (namely, p.L483P, p.A448H, complex mutations, R285H, G202R, and W184R) in almost all individuals might explain the high prevalence of neurological and sensory abnormalities observed in our patients diagnosed with GD1 [20,40,41,42,43]. The same prevalence could also be partially explained by the significantly higher age at evaluation in GD1 patients. Indeed, GBA mutations have been considered as a paradigmatic example for studying neurodegenerative processes [44], and we could speculate that GBA mutations may be associated with an accelerated aging process, leading to progressive neurological impairment during adulthood and old age.

## 4. Materials and Methods

### 4.1. Patients

This prospective observational study, named SENOPRO, included 22 GD patients (19 GD1 and 3 GD3), either naïve to therapy or receiving ERT, referred to the AOU Policlinico Umberto I. Inclusion criteria included a genetically confirmed diagnosis of GD and age greater than 12. According to current clinical practice, we planned to perform multidisciplinary evaluations, including MRI 3Tesla (Table 3) at baseline and, thereafter, approximately 12 months and 24 months (current practice for follow-up) and/or in case of the onset of neurological symptoms. Neurological, neuroradiological, neuropsychological, ophthalmological, and hearing assessments were performed on each patient over 3–4 consecutive days. According to the observational design of this study, functional and ultrastructural data were obtained without undertaking further examinations or prolonging the time dedicated for the requested assessments. This study was approved by the local ethics committee.

### 4.2. Neurological and Neuroradiological Assessment

All patients were interviewed by a trained neurologist to ascertain the possible occurrence of sleep disturbances, namely, restless leg syndrome and rapid-eye-movement (REM) behavior disorder, according to well-established criteria [45,46]. The ESS was used to assess EDS and a score > 10 was considered as a positive diagnosis of EDS [47]. The occurrence of parkinsonian motor signs (namely, bradykinesia, rigidity, and tremor) was independently assessed in each patient by two neurologists (CI, CDB), and the MDS-UPDRS III was administered to all patients [48]. To assess parkinsonian NMS, the NMSS was used [49]. NMSS explores 30 different NMS, grouped into 9 different domains: cardiovascular autonomic instability, sleepiness/fatigue, mood/apathy, perceptual problems, memory/attention, gastrointestinal disturbances, urinary tract dysfunction, sexual activity, miscellaneous (i.e., pain not related to known medical conditions, hyposmia, sweating disturbances, and unexplained weight changes). All patients underwent a video-EEG exam using a Micromed System Plus 21-channel device, including common activation procedures (e.g., intermittent photic stimulation and hyperventilation). EEG electrodes were placed on the scalp according to the 10–20 International System. Finally, MRI data were collected on a 3T Siemens MAGNETOM Verio scanner with a twelve-channel phase-array head coil. Nineteen subjects (17 GD1, 2 GD3, median age = 44, range 17–68 years), underwent a 3T Magnetic Resonance Imaging session including T1, PD-T2 weighted sequences, Fluid Attenuated Inversion Recovery (FLAIR), and Diffusion Tensor Imaging (DTI) sequences. All the parameters of the MRI sequences are reported in the Supplementary Materials.

Based on previously published data indicating selective astrogliosis in the hippocampus in GD patients [10] we performed a brain morphometry analysis of this brain structure in our cohort of patients and in healthy subjects, previously acquired and selected to be comparable in terms of age (see Section 4.6).

### 4.3. Neuropsychological Assessment

The MMSE [50] was used to measure global cognitive decline. In addition, patients underwent an extensive neuropsychological evaluation consisting of Rey’s auditory verbal learning test [51], immediate visual memory [51], Corsi block-tapping test [52], Digit Span [52], Babcock story for the memory domain [53]; multiple features target cancellation (Toulouse-Piéron) test [54] and trail-making test for the attentional domain [55]; word fluency for language and executive functions [51], phrase construction for language [51]; freehand copying of drawings and copying drawings with landmarks for visuo-constructional skills [51]; and Raven’s progressive colored matrices for abstract reasoning abilities [51]. Moreover, each participant was interviewed and evaluated with the BPRS before the neuropsychological assessment [56].

### 4.4. Hearing Evaluation

ABR was used to evaluate the involvement of the auditory pathway and was obtained while the subject was sitting comfortably in a soundproof room. Recording was performed with an Interacoustics Eclipse EP25 (Interacoustics AS, Drejervaenget 8, DK-5610 Assens, Denmark) with a 21-stimuli-per-second click rate, in an analysis window of 12 ms. Further details regarding ABR are explained in the Supplementary Materials. Assessment of PTA at octave frequencies between 125 and 8000 Hz was performed using frequency-modulated tones in a standard soundproofed booth. Assessment was performed through an auricle audiometer (Otometrics, Taastrup, Denmark) connected to TDH39 headphones (see Supplementary Materials). Speech perception in quiet was assessed with balanced sentence lists from the Italian Speech Audiometry [57], with the speech signal at 65 dB SPL presented at 0° to the participant’s head. The score ranges from 0 to 100%. Speech perception in noise was evaluated using the Matrix tests [58] adapted in Italian (see Supplementary Materials).

### 4.5. Ophthalmological Evaluation

All participants underwent a medical history collection. A complete ophthalmological examination included an orthoptic visit, a slit lamp exam, an assessment of BCVA measured using the early treatment diabetic retinopathy study (ETDRS) charts at 4 m, an IOP, a pattern-reversal VEPs, a flash standard full-field ERG, and a SD-OCT. See the Supplementary Materials for a detailed description of the acquisition parameters for VEPs, ERG, and SD-OCT.

### 4.6. Statistical Analysis

Data were tested for normal distribution using data visualization methods and the Shapiro–Wilk test. Mean and median were presented according to normal, or non-normal, distribution of data. Comparison across groups was performed with the student t-test, or the Mann–Whitney U test/Wilcoxon test. Correlations between continuous variables were examined using Spearman’s Rank correlation coefficient technique, whereas the chi-square test was used for nominal variables. *p* values < 0.05 were considered statistically significant. Analyses were performed using SPSS version 27.

To measure the GM volume of the left and right hippocampus in T1 structural images of both GD patients and healthy volunteers, we used the Computational Anatomy Toolbox (CAT12) software, which is implemented within the Statistical Parametric Mapping (SPM12) software package. Imaging data were preprocessed and analyzed using Voxel-based morphometry (VBM). The region of interest (ROI)-based value of GM volume was estimated in native space before any spatial normalization, according to the neuromorphometric atlas. Further details regarding VBM are explained in the Supplementary Materials.

To examine whether there was a significant difference in hippocampal grey matter volume between patients with GD and healthy controls, we performed an analysis of covariance (ANCOVA) while controlling for age and total intracranial volume (TIV). Furthermore, we conducted a one-tailed partial correlation analysis using the Spearman’s rank correlation coefficient to examine the association between the performance on the neuropsychological tests and the hippocampal GM volume (left and right) in GD patients, while adjusting for the potential confounding effects of age, schooling, and total intracranial volume (TIV). The statistical analyses were performed using Jamovi (https://www.jamovi.org, 10 May 2023), an open-source software package for statistical analysis and data visualization.

## 5. Conclusions

Following our multidisciplinary study, we highlight our relevant findings: (1) a high rate of parkinsonian motor and non-motor symptoms, especially in GD1 patients harboring severe GBA variants; (2) a frequent occurrence of various degrees of cognitive impairment, both in patients initially classified as GD1 and GD3; (3) a significant correlation between left hippocampal brain volume and impaired short- and long-term performance; (4) an impaired speech perception in noise in the majority of patients, indicative of impaired central processing of hearing and associated with high rates of slight hearing loss both in GD1 and GD3; (5) relevant structural and functional abnormalities along the visual system, both in GD1 and GD3. The high prevalence of sleep disturbances detected in our patients led us to systematically include dedicated polysomnographic studies on the scheduled SENOPRO timeline, with the aim of better understanding the pathophysiology of sleepiness in GD patients. We also plan to correlate extensive brain morphometry and functional connectivity in GD patients by using suitable fMRI data analysis.

Overall, our findings further support the concept of GD as a continuum of disease subtypes, especially in terms of neurological involvement, and reveals, once again, the thin line and overlapping borders existing between different GD subtypes. Our data support the need for in-depth periodic monitoring of cognitive and motor performances, mood, behavior, and sleep patterns in all patients with GD, independently from the patient’s initial classification. Future prospective studies should assess the prognostic impact of some of the detected biomarkers for subsequent neurodegeneration.

## Figures and Tables

**Table 1:** Characteristics of the 22 GD enrolled patients. Abbreviations: ERT = enzyme replacement therapy; GD = Gaucher disease; SRT = substrate replacement therapy.

Features	All GD Patients	GD1 Patients	GD3 Patients
**Total number**	22	19	3
**Gender**			
Male	9 (41%)	6 (31.6%)	3
Female	13 (59%)	13 (68.4%)	-
**Genotyping**			
p.A409S/p.L483P	8 (36%)	8 (42%)	-
p.A409S/recNCiI	2 (9%)	2 (10%)	-
p.A409S/P138LFs*62	2 (9%)	2 (10%)	-
p.A409S/c.1389-1G>A	1 (4%)	1 (5%)	-
p.A409S/complexI	1 (4%)	1 (5%)	-
p.A409S/2123insA	1 (4%)	1 (5%)	-
p.A409S/R285H	1 (4%)	1 (5%)	-
p.A409S/W184R	1 (4%)	1 (5%)	-
p.A409S/p.G241A	1 (4%)	1 (5%)	-
p.L483P+V460V/RecFs	1 (4%)	1 (5%)	-
p.L483P/p.L483P	2 (9%)	-	2 (66%)
p.A448H/p.A448H	1 (4%)	-	1 (33%)
**Median age at diagnosis (years)**	20.19	20.19	1.3; 11.67; 17
**(range)**	(1.3–46.58)	(4.24–46.58)	
**Median age at the time of study (years)**	44.51	44.87	17.5; 26.33; 33.85
**(range)**	(17.15–67.92)	(18.3–67.92)	
**Neurological symptoms at diagnosis**	4 (18%)	1 (5%)	3
Psycho-cognitive disturbances	1	1	-
Eye movement abnormalities	3	-	3
**Treatment**	21 (95.5%)	18 (95%)	3
ERT	17	10	3
SRT	3	8	-
None	1	1	-
**Median treatment duration (years)**	17.32	18.57	8.43, 15.85, 21.58
**(range)**	(1.57–26.87)	(1.57–26.87)	

**Table 2:** Neurological and sensory abnormalities in each patient at baseline evaluation.

			Neurological Assessment			Neuropsychological Assessment		MRI	Opthalmological	Hearing
Pt	Genotype	Age/Sex	Parkinsonism	Other Motor Signs	Sleep	Cognitive	Psychiatric			
**GD TYPE 1**										
#01	p.A409S/p.L483P	33/M	Bradykinesia		EDS				VEP: reduced amplitude, and increased latency	
#02	p.A409S/p.L483P	55/M						n/a	VEP: reduced amplitude, and increased latency	Slight SNHL
#03	p.A409S/p.L483P	45/F	Bradykinesia; rigidity		RLS	Cognitive impairment	Psychosis			Slight SNHL
#04	p.A409S/p.L483P	45/F	Bradykinesia; rigidity, rest tremor		EDS; RBD	Mild cognitive impairment	Anxiety; Depression Somatic concerns	n/a	VEP: reduced amplitude	Cookie and reverse cookie slight SNHL
#05	p.A409S/p.L483P	28/F	Bradykinesia		EDS					Slight SNHL
#06	p.A409S/p.L483P	53/F	Bradykinesia		EDS; RLS					Slight SNHL
#07	p.A409S/p.L483P	39/F			RLS		Anxiety	Subcortical areas of gliosis	VEP: reduced amplitude; SD-OCT: retinal thinning	
#08	p.A409S/p.L483P	41/M					Anxiety; Depression	Developmental venous anomaly	SD-OCT: retinal thinning	Slight SNHL
#09	p.A409S/recNCiI	58/M	Bradykinesia; rigidity		EDS				VEP: reduced amplitude, and increased latency	Cookie and reverse cookie slight SNHL
#10	p.A409S/recNCiI	51/F			EDS; RBD		Anxiety		VEP: reduced amplitude	Cookie and reverse cookie slight SNHL
#11	p.A409S/P138LFs*62	18/F				Mild cognitive impairment	Anxiety, Somatic concerns			
#12	p.A409S /P138LFs*62	18/M							SD-OCT: retinal thinning	
#13	p p.A409S /c.1389- 1G>A	51/F					Somatic concerns	Dilation of perivascular spaces in the sub-cortical and nucleus-basal area	VEP: reduced amplitude	Slight SNHL
#14	p.A409S/complexI	57/F			RLS	Mild cognitive impairment		Subcortical areas of gliosis	VEP: reduced amplitude SD-OCT: retinal thinning, and macular alterations	Slight SNHL
#15	p.A409S/2123insA	58/F	Bradykinesia			Cognitive impairment		Subcortical areas of gliosis	VEP: increased latency; SD-OCT: retinal thinning	Slight SNHL
#16	p.A409S/R285H	68/F			EDS; RLS			Diffuse suffering of white brain matter due to chronic ischemic vascular damage		Slight SNHL
#17	p.A409S /W184R	44/F					Somatic concerns		SD-OCT: retinal thinning	Cookie and reverse cookie slight SNHL
#18	p.A409S/p.Gly241Arg	44/F			RBD		Depression			
#19	p.L483P+V460V/RecFs	32/M			Central hypersomni a		Somatic concerns	Enlargement of posterior horns of lateral ventricles		
**GD TYPE 3**										
#20	p.L483P/p.L483P	26/M		Mild pyramidal signs; saccadic impairment		Mild cognitive impairment		Enlargement of posterior horns of lateral ventricles	VEP: reduced amplitude	SNHL of variable shape
#21	p.L483P/ p.L483P	17/M		Saccadic impairment					VEP increased latency	SNHL of variable shape
#22	p.A448H/p.A448H	34/M		Weakness; fascicul ation; saccadic impairment	EDS	Mild cognitive impairment	Somatic concerns	n/a	VEP: increased latency; SD-OCT: retinal thinning, and macular alterations	SNHL of variable shape

Abbreviations: EDS = excessive daytime sleepiness; RBD = REM behavior disorder; RLS = restless leg syndrome; SD-OCT = spectral domain optical coherence tomography; SNHL = sensorineural hearing loss; VEP = visual evoked potential; n/a = not acquired.

**Table 3:** Detailed multidisciplinary investigations provided for the SENOPRO study.

**Neurological Assessment**
Clinical evaluation with ocular motility exam Severity Scoring Tool, Unified Parkinson’s Disease Rating scale and Epworth Sleepiness scale, non-motor-symptom scale (NMSS)
Electroencephalography (EEG)
**Psycho-Diagnostic And Psychiatric Evaluation**
Test battery (Corsi test, Barrage test, Mini-Mental State Examination, Mental Deterioration Battery, and Trail Making Test)
Brief Psychiatric Rating Scale (BPRS)
Cognitive Behavioral Assessment (CBA 2.0)
**Ophtalmological Evaluation**
Physical evaluation
Pattern visual evoked potentials (VEPs)
Full-field electroretinogram exam (ERG)
Spectral Domain-Optical Coherence Tomography (SD-OCT)
**Auditory Evaluation**
Auditory Brainstem Response (ABR)
Pure tone audiometry (PTA) at octave frequencies between 125 and 8000 Hz
Speech perception in quiet and in noise using Matrix tests
**Neuroimaging**
Magnetic Resonance (MR 3Tesla)

## Data Availability

Data are available upon request to the corresponding author in accordance with the institutional ethics approval.

## Supplementary Materials

The following supporting information can be downloaded at: https://www.mdpi.com/article/10.3390/ijms24108844/s1.
