# Supplementary material for: The Spectrum of Neurological and Sensory Abnormalities in Gaucher Disease Patients: A Multidisciplinary Study (SENOPRO)
Source: Int J Mol Sci. 2023 May 16;24(10):8844. doi: 10.3390/ijms24108844 (PMC10218502; doi:10.3390/ijms24108844)
Supplement: Supplementary file 1 [file ijms-24-08844-s001.zip › ijms-2382456-supplementary.pdf]

Supplementary materials

Materials and methods

1. Neuroradiological assessment

The 3T Magnetic Resonance Imaging (3T MRI) session included the following sequences: T1 (TR= 1.9s, TE=2.93s, flip angle =9°, voxel size: 1.0×1.0×1.0 mm), PD-T2 weighted sequences (TR=3s, TE1=9.4 ms, TE2= 94 ms, voxel size= 0.7x0.7x3.0 mm), FLAIR (TR=9s, TE=94 ms, flip angle= 150°, voxel size= 0.9x0.9x3.0 mm), Diffusion Tensor Imaging (DTI) (TR=4.6s,TE=78 ms, flip angle=90°,voxel size=2x2x2 mm).

1.1 Voxel- and ROI-based Morphometry analysis

To measure the grey matter (GM) volume of the left and right hippocampus in T1 structural images of both GD patients and healthy volunteers we used the Computational Anatomy Toolbox (CAT12) software, which is implemented within the Statistical Parametric Mapping (SPM12) software package (www.fil.ion.ucl.ac.uk). Imaging data were preprocessed and analyzed using the Voxel-based morphometry (VBM) which is a fully automated technique for computational analysis of differences in regional grey matter (GM), white matter (WM) or cerebral spinal fluid (CSF) volume. Images were first segmented into GM, WM, and CSF, and then Region of interest (ROI) -based values of GM volume were estimated in native space before any spatial normalization, according to the neuromorphometric atlas. For each patient and healthy volunteer we included in the analysis the gray matter volume values from hippocampus, ROI included in the neuromorphometric atlas.

1.1 Hearing evaluation

Auditory brainstem recordings (ABR) were used to evaluate the involvement of the auditory pathway in GD patients. The highest intensity reached was 100 dB-hearing level (dB-HL). The test implicates the repetition of the stimulus by 10 dB-HL steps, and the lowest intensity at which the V wave is observed represents the hearing threshold of the subject. The evaluation criterion only included the presence of the wave V, and latency and amplitude values were recorded (V wave latency considered normal for values between 5.20 to 5.70 ms at 80 dB HL).

Pure Tone Audiometry (PTA). All patients were tested for assessment of PTA at octave frequencies between 125 and 8000 Hz, using frequency-modulated tones in a standard soundproofed booth. Assessment was performed through an Aurical audiometer (Otometrics Taastrup, Denmark) connected to TDH39 headphones. Speech and noise stimuli were presented via a computer and a preamplifier connected directly to a single loudspeaker which was placed at 0 azimuth and at 1m distance from the participant's head.

Speech perception. Speech perception in quiet was assessed with the balanced sentence lists from the Italian Speech Audiometry [57], and with the speech signal presented at 0° from the participant's head at 65 dB SPL. The range of scores is from 0-100%. Speech perception in noise was evaluated using the Matrix tests [58] adapted in Italian. This test can be used for repeated accurate measurements with the same listener, as they use semantically unpredictable sentences with a fixed syntactic structure and a random selection of items. The Matrix test is a closed-set test in nature, due to a limited number of item options. However, in contrast to everyday sentences, those generated in the Matrix test have low semantic predictability. Because of the semantically unpredictable structure, the lists cannot be memorized easily and thus, can be used repeatedly. According to the number of correctly understood words in the preceding sentence, the software adapts the speech level for the following sentence, and starting at 0 dB SNR, the procedure iterates an estimate of the SNR level where 50% of presented words are understood (Speech Reception Threshold, SRT).

The SRT was obtained with noise fixed at 65 dB SPL and the signal adaptively changed to obtain the SRT value. In this study, assessment was performed with an open-set response. Each test list contained 30 sentences and was preceded by two training lists. The mean SRT of a normal hearing subject was -7.3 ± 0.2 dB signal/noise ratio (dB SNR). The clinical value difference in matrix was 1 dB SNR.

1.1 Ophthalmological evaluation

The study of bioelectric conduction of visual pathways was carried out using transient VEPs. The transient VEP was performed at a distance of 100 cm from the optoelectronic stimulator Vision Monitor MonPack 120 by Metrovision (Pérenchies France) according to the International Society for Clinical Electrophysiology of Vision guidelines: stimulus field size 23.6 arc deg centered on the fovea, contrast 100%, mean luminance 50 cd/m2, and 2 reversals/s. The VEPs were elicited by checkerboard stimuli with large 120' (min arc), medium 60' (min arc), and small 15'(min arc) checks. The waveform of the transient examination was obtained at low temporal frequencies of the stimuli. The values of the amplitude and latency (or time-to-peak) of P100 peaks were then studied. The standard VEP protocols were coded for a single recording channel with a midline occipital active electrode, a referent electrode on the frontal region of the face, and a neutral electrode on the earlobe. VEP was identified by a series of N75, P100, and N135 peaks, each characterized by amplitude and latency.

A standard ganzfeld full field ERG was performed after the VEP exam using the computerized Optoelectronic Stimulator Vision Monitor Mon-Pack 120 Metrovision (Pérenchies, France), according to the ISCEV guidelines (International Society for Clinical Electrophysiology of Vision). Before recording, the pupils were dilated with topical tropicamide 1%. All the subjects were dark adapted for 30 minutes. The corneas were then anaesthetized with topical proparacaine hydrochloride 0.5 per cent. The ERG recording was performed using an ERG-Jet corneal contact lens active electrode. A skin reference, or inactive electrode was attached to the outer corners of both eyes, and a ground electrode was put on the patient's earlobe. The active, inactive, and ground electrodes were connected to a junctional box from which the signals were delivered to additional recording components for amplification and display.

All patients were evaluated by SD-OCT, a non-contact imaging technology that provides a detailed cross-sectional view of the retinal profile, and the optic disks, using an infrared laser probe and confocal scanning laser ophthalmoscopy. SD-OCT data was obtained using the Spectralis OCT (Spectralis® HRA/OCT Heidelberg Engineering, Heidelberg Germany). A macular thickness map was obtained for each eye, using the raster 25° X 15°, 25-line horizontal raster scan protocol, centered on the fovea, with up to 10 frames averaged for each scan. Retinal thickness and optic disks were automatically calculated by the software.
